# Asbestos Exposure Level and the Carcinogenic Risk Due to Corrugated Asbestos-Cement Slate Roofs in Korea

**DOI:** 10.3390/ijerph18136925

**Published:** 2021-06-28

**Authors:** Eun-Soo Lee, Young-Ki Kim

**Affiliations:** 1 Department of Occupational and Environmental Medicine, Pusan National University Yangsan Hospital, Yangsan 50612, Korea; es3003@pnuyh.co.kr; 2 Department of Preventive and Occupational & Environmental Medicine, School of Medicine, Pusan National University, Yangsan 50612, Korea; 3 Environmental Health Center of Asbestos, Pusan National University Yangsan Hospital, Yangsan 50612, Korea

**Keywords:** asbestos, exposure, cancer, risk

## Abstract

Asbestos-cement slate roofs are one of the most common environmental causes of asbestos exposure. However, few studies have examined residential asbestos-cement slate-related exposure and its effects on human health. This study was performed to evaluate cumulative asbestos exposure levels and to calculate the Excess Lifetime Cancer Risk (ELCR) of residents of asbestos-cement slate-roofed houses. We reviewed previous Korean literature to estimate the concentration of airborne asbestos from asbestos-cement slate roofed buildings. Finally, eight studies were selected, and a pooled analysis was performed. The results derived from the pooled analysis were combined with the data from a health impact survey conducted from 2009 to 2016 at the Environmental Health Center for Asbestos (EHCA) of the Yangsan Pusan National University Hospital, and a carcinogenic risk assessment was performed. As a result, the representative value of the indoor exposure concentration related to asbestos-cement slate was found to be 0.0032 f/cc on average, and the representative value of the exposure related to occupational asbestos-cement slate dismantling and demolition was found to be 0.0034 f/cc. In addition, the ELCR of asbestos-cement slate related indoor exposure and occupational dismantling and demolition was found to be of medium risk, and the ELCR of residential dismantling and demolition of asbestos-cement slate was less than 10^−6^, indicating that the risk was low. Since there is no threshold for carcinogenicity related to asbestos, this should not be ignored even if the risk appears low, and it would be reasonable to calculate the carcinogenic risk based on total lifetime exposure. More studies on asbestos exposure scenarios and the scope of similar exposure groups through additional data collection and further analysis of risk are needed.

## 1. Introduction

Asbestos is a natural fibrous silicate mineral belonging to the group of serpentine and amphibole minerals, and has unique advantages such as low price, flexibility, non-flammability, insulation, acid, and alkali resistance. For this reason, it has been used worldwide in various industries such as construction materials (e.g., cement roofing sheets, plumbing, and ceiling materials), automobile products (e.g., brake linings), and textile products. As per the legal definition in European countries, asbestos is made up of fibers with a length >5 µm, width <3 µm, and more than 3:1 aspect ratio [1,2]. In Korea, 145,533 tons of asbestos were produced at asbestos mines such as the Gwangcheon asbestos mine located in Hongseong, Chungcheongnam-do, and it is estimated that a total of 1,697,477 tons of asbestos have been imported and used since 1976 [3]. However, as the risk of asbestos-related diseases (ARDs) such as asbestosis, lung cancer (LC), and malignant mesothelioma (MM) was recognized, the Occupational Safety and Health Act (OSHA) was revised in February 2009, and subsequently, the import, distribution, and use of asbestos were completely prohibited.

**Figure 1:**
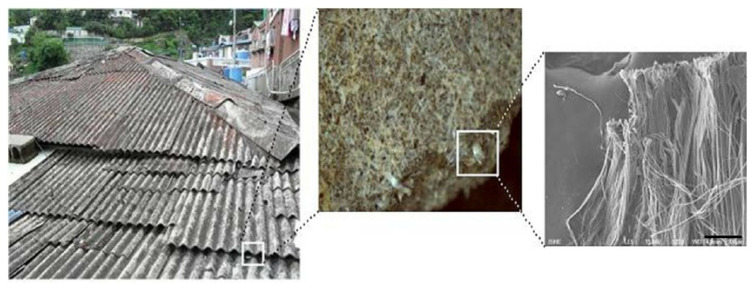
Asbestos-cement slate roofs (**left**) and Scanning Electron Microscope (SEM) image (**right**) of chrysotile in the slate roofs. The bar size in the right image indicates 100 μm.

Asbestos-cement slate roofs are produced by adding 10–20% chrysotile to 80–90% cement (Figure 1) [4,5]. In Korea, as part of the Saemaul Undong (New Village Movement), the Korean government led a project to improve the roofs in rural areas in the late 1960s, and extensively distributed asbestos-cement slate as a roofing material. In the 1970s, most of the asbestos imports were used for asbestos-cement slate, and it is estimated that 96% of the asbestos in Korea was used for asbestos-cement slate manufacturing [6]. Asbestos-cement slate was mainly produced by the wet process, which results in a relatively low level of asbestos exposure. However, the problem of exposure to asbestos in the process of natural weathering, repair, or demolition of buildings using an asbestos-cement slate roof was still significant. Therefore, the production of asbestos-cement slate was stopped with the revision of the Enforcement Decree of the Industrial Safety and Health Act in September 2006. Nevertheless, according to a survey conducted by the Ministry of Environment in 2008, about 1.23 million asbestos-cement slate buildings existed across Korea, of which 660,000 buildings were in urban areas and 570,000 in rural areas. Most of these (878,000 buildings, 71.4%) were residential buildings. Furthermore, as buildings constructed before the 1970s accounted for more than half (55.4%) of the total, there was concern that the scattering of asbestos from these aging buildings would increase [7]. Currently, in Korea, compensation has been provided for asbestos-related diseases caused by environmental exposure to asbestos. Therefore, diseases caused by exposure to asbestos from an asbestos-cement slate roof are also subject to compensation. Hence, exposure data and criteria to evaluate the impact on health are required to determine the exposure intensity and carcinogenic risk. Previously, there have been studies to measure the exposure level of airborne asbestos from asbestos-cement slate-roofed buildings, but the measurement conditions and results showed significant variations, making it difficult to determine a specific value to represent the degree of exposure [8]. Only a few studies have attempted to calculate the carcinogenic risk from this exposure [5,9]. Given the paucity of data, we calculated a representative value of the exposure concentration of asbestos in an asbestos-cement slate-roofed house by analyzing the results of past studies to propose a standard value for the asbestos injury relief system and analyzed the carcinogenic risk due to exposure to asbestos-cement slate roofs. In addition to indoor asbestos exposure scenarios for houses with asbestos-cement slate roofs, we analyzed the cumulative asbestos exposure levels and carcinogenic risk of an additional scenario when residents were repairing or dismantling their own asbestos-cement slate roof. This is because residents of asbestos-cement slate roofs often make repairs or replace them on their own when roofs are broken. This releases much higher concentrations of asbestos dust than typical situations of indoor exposure.

## 2. Materials and Methods

### 2.1. Information Sources and Search

To estimate the concentration of airborne asbestos from Korean asbestos-cement slate-roofed buildings, the literature in the Korean database was reviewed. The search word used was “asbestos slate”, and documents published in Research Information Sharing Service (RISS, Daegu, Korea), Korea Citation Index (KIC, Daejeon, Korea), and Google Scholar until December 2019 were included. However, all the articles searched in KCI overlapped with those searched in RISS, therefore only the results searched in RISS were considered.

### 2.2. Inclusion and Exclusion Criteria

The literature search included all thesis and research reports published in Korean journals. When a master’s article was published in a journal, the article published in the peer-reviewed journal was selected. However, if the research was the same, but the required results were not available in the published article, the master’s article was also cited. All the articles identified in the initial search were first screened by title, and then by abstract. Finally, the entire text was reviewed and selections were made.

**Figure 2:**
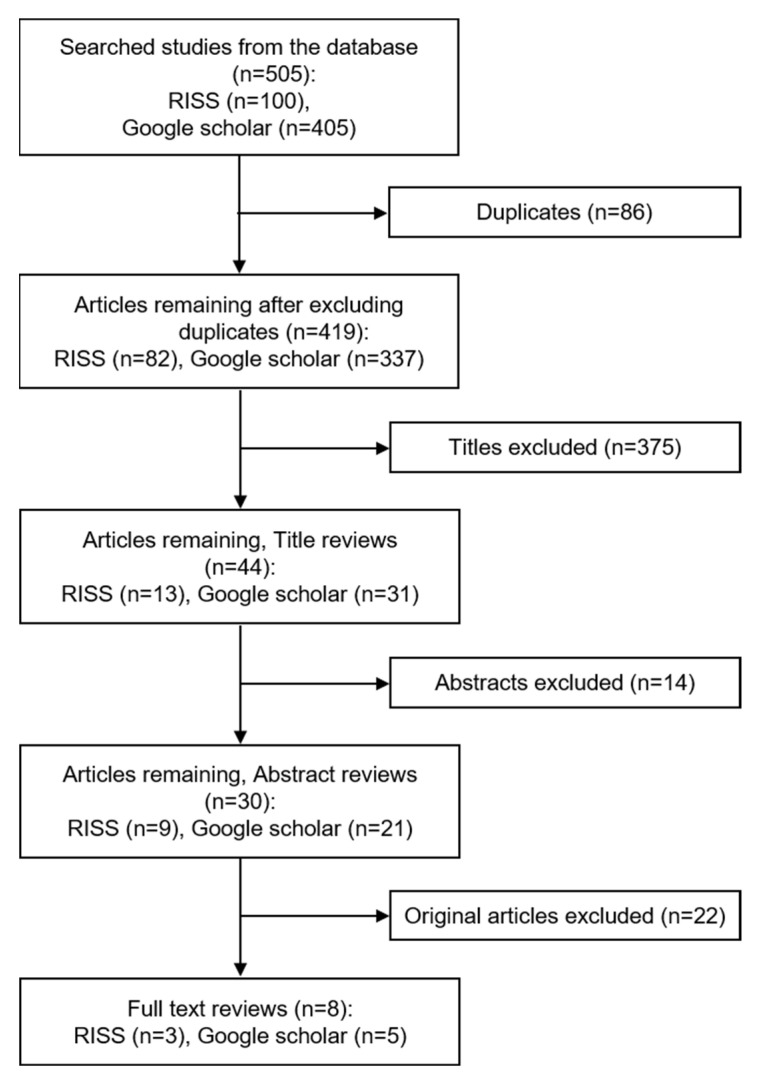
Flow of the Selection Process of Studies for Review.

When the asbestos concentration value was not expressed as f/cc or f/mL, it was excluded from the final analysis, and articles showing the mean, minimum and maximum values but not indicating standard deviation were also excluded (Figure 2).

### 2.3. Data Extraction and Pooled Analysis

Finally, eight studies were selected and classified into similar exposure groups and a pooled analysis was performed. In each paper, the number of samples, geometric mean and standard deviation, arithmetic mean and standard deviation, minimum and maximum values, analysis methods, the year of establishment of the asbestos-cement slate building, and specifics for each exposure scenario were extracted. Exposure was classified by building establishment year (before 1980, after 1980), indoor and outdoor exposure at asbestos-cement slate buildings, and exposure during dismantling and demolition (applies to repairs) (Table 1).

The final results were expressed as the arithmetic mean and the arithmetic standard deviation, and if the results of original articles were displayed as geometric mean and geometric standard deviation, the result was converted to the arithmetic mean under the assumption of a log-normal distribution.

### 2.4. Cancer Risk Assessment

The results derived from the pooled analysis were combined with the data from a health impact survey conducted from 2009 to 2016 at the Environmental Health Center for Asbestos (EHCA) of the Yangsan Pusan National University Hospital, South Korea, and a carcinogenic risk assessment was performed [16]. Out of a total of 11,191 subjects, 1228 people were classified into an asbestos-cement slate exposed group. These were people who responded that they had been living in asbestos-cement slate-roofed houses at the time of the survey or who were proven to have lived in an asbestos-cement slate-dense area through the certificate of resident register. We reclassified the asbestos-cement slate-exposed group into the “only slate-exposed group (*n* = 575)” with no occupational, family, or other environmental asbestos exposure. We performed an analysis of the characteristics of the exposed group. The cumulative asbestos exposure concentration and the Excess Lifetime Cancer Risk (ELCR) in the “only slate-exposed group” were calculated to evaluate the asbestos-cement slate related carcinogenic risk for these subjects. This method was adopted to evaluate the effect of asbestos-cement slate exposure alone.

The carcinogenic risk was calculated based on the Guidelines for Carcinogen Risk Assessment proposed by the US Environmental Protection Agency (EPA) [17]. The equation used for estimating risks from asbestos inhalation was:

ELCR = EPC × TWF × IUR

where ELCR = Excess Lifetime Cancer Risk, EPC = Exposure Point Concentration, TWF = Time Weighting Factor), and IUR = Inhalation Unit Risk.

For the EPC, the concentration obtained from the pooled analysis for each scenario was applied. In the calculation of TWF, the exposure time was calculated by applying the average time spent indoors by Korean adults over 19 years of age as 14.90 h on weekdays and 16.46 h on weekends [18]. The weekdays per year were calculated as 260 days (52 weeks × 5 days) and weekends per year were calculated as 104 days (52 weeks × 2 days), and holidays were not considered. Inhalation Unit Risk (IUR) used the values suggested by EPA’s Integrated Risk Information System (IRIS) [17,19,20,21]. However, since there was no mention of the timing of the first exposure to the asbestos-cement slate in the original data, the age at the time of examination minus the first residence age in the asbestos-cement slate area was determined as the asbestos-cement slate exposure period. In some cases, the IUR value could not be found in the EPA’s IUR table. Here, the age at onset value was inferred and substituted into the calculation formula through the ten-year trend. Similarly, when the exposure period was not in the original data, the average value was calculated (i.e., when the case’s exposure period is 15 years, only the IUR values of 14 and 16 years are displayed in the EPA’s IUR table, so the average of the two values is calculated and included).

In the case of repair, dismantling, and demolition of asbestos-cement slate roofs by the residents, the results of occupational dismantling and demolition were applied as there was no related research content.

In the health impact survey, the most frequent answer to the query on the period of asbestos-cement slate exposure was more than 20 years, and the most frequent answer to asbestos-cement slate repair period was less than 4 years. Therefore, we assumed that the roof was repaired once every 5 years, 8 h a day. Therefore, under the assumption that the occupational dismantling and demolition is done 5 days a week, 8 h a day, we calculated the frequency of dismantling and demolition at the residences as follows; 1/(5 years × 52 weeks × 5/7 days) of the occupational dismantling and demolition = 1/185.7 (about 0.5%).

### 2.5. Statistical Analysis

The statistical analysis and data trimming were performed using SAS 9.4 (SAS Institute, Cary, NC, USA) and Microsoft Excel 2016 (Microsoft Inc., Redmond, WA, USA).

## 3. Results

The results of the pooled analysis of asbestos exposure concentrations according to each scenario are expressed as an arithmetic mean and standard deviation (Table 2). The asbestos exposure level of residential asbestos-cement slate roof repair or dismantling could not be analyzed as no related research was found, so we estimated the same from the results of occupational dismantling and demolition.

Table 3 presents the characteristics of the asbestos-cement slate-exposed group. Of the total of 1228 subjects, it was estimated that 75.65% of the cases lived only under asbestos-cement slate roofs, and 24.35% lived both under asbestos-cement slate roofs and in asbestos-cement slate-dense areas. The highest living period exposed to asbestos-cement slate was 60.34% of cases for more than 20 years, followed by 10 to 19 years, 5 to 9 years, and less than 4 years. In addition, 362 respondents (29.48%) answered that they had carried out asbestos-cement slate roof repairs, and 200 people with the most exposure had carried out these repairs for less than 4 years.

Next, we calculated the cumulative exposure concentration of asbestos and the ELCR for the “only slate-exposed group” (Table 4 and Table 5).

The total cumulative exposure concentration of asbestos for indoor exposure was 0.044 fiber-years/cc on average, and in the case of occupational asbestos-cement slate dismantling and demolition, the total cumulative exposure concentration of asbestos was 0.047 fiber-years/cc. The calculated cumulative exposure concentration of dismantling and demolition of asbestos-cement slate roofs in residents was 0.000251 fiber-years/cc on average.

The total ELCR for indoor exposure of the only-slate exposed group was 4.81 × 10^−5^ on average (4.81 carcinogenicity per 100,000 people), and the total ELCR for the dismantling and demolition of the asbestos-cement slate exposed group was on average 5.11 × 10^−5^ (5.11 carcinogenicity per 100,000 people). The calculated ELCR for residents involved in the dismantling and demolition of the asbestos-cement slate was 2.75 × 10^−7^ (2.75 carcinogenicity per 10,000,000 people).

When the ELCR of the indoor exposure group was divided into three groups according to the risk, 89.22% were categorized as intermediate-risk (10^−6^ ≤ ELCR < 10^−5^) (Table 6).

## 4. Discussion

Asbestos-cement slate roofs are a major source of environmental asbestos exposure along with asbestos mines and factories. However, this exposure type is under-researched as compared to other sources of exposure. Therefore, there are many difficulties in evaluating its health risk.

In this study, through a literature review and pooled analysis, the representative value of asbestos exposure concentration in residents of an asbestos-cement slate roof house was postulated for the first time in Korea. The carcinogenic risk of each exposure scenario was calculated so that it could be used to establish a standard for objectively and quantitatively evaluating the intensity of asbestos-cement slate-related asbestos exposure in asbestos-related disease patients.

Until now, the amount of asbestos exposure due to asbestos-cement slate roofs has been measured, but only one-time measurements have been taken, and no studies have estimated the cumulative exposure. In our study, the cumulative exposure concentration of asbestos in asbestos-cement slate roof house occupants through pooled analysis was calculated at 0.044 fiber-year/cc on average for indoor exposure, and 0.047 fiber-year/cc for occupational asbestos-cement slate roof dismantling and demolition. The cumulative exposure concentration due to dismantling/demolition of asbestos in residents was 0.00025 fiber-year/cc. In all cases, there was a tendency of cumulative exposure concentration to increase with age and there was no variation according to sex.

A few studies have reported the effects of exposure to residential asbestos-cement slate roofs on health. In a previous study using the same data as this study, the odds ratio (OR) value of lower lung fibrosis was 5.5 (3.3~9.0), pleural disease was 8.8 (5.6~13.8), lung mass was 20.5 (10.4~40.4) in residents of asbestos-cement slate roof houses and living in asbestos-cement slate dense areas [16].

Most of the foreign studies have been concerned with occupational asbestos-cement slate-related carcinogenic risk or asbestos exposure levels. In the case of foreign countries, the tendency to use the term “asbestos cement sheet” rather than “slate roof” was confirmed. In a Thai study, the relative risk of lung cancer was confirmed by calculating the cumulative exposure from asbestos cement roof sheet work. The cumulative asbestos exposure concentration estimated through existing literature and the past nationwide air sampling concentration was 90.13~115.65 fiber-years/mL, and the relative risk (RR) of lung cancer calculated by the EPA model was 5.37~5.96 [22].

In another study, asbestos concentration levels and carcinogenic risk were confirmed by examining the airborne concentration of asbestos at the time of construction or repair of roof tiles in Thailand. As a result of the study, the airborne concentration of asbestos ranged from 0.02 to 0.1 fibers/cm^3^, and when using EPA’s mathematical model, the level of exposure to asbestos emitted from the roof tiles would result in not greater than a “one-in-twenty” to “one-in-four” increased chance of developing cancer [23].

A Polish study measured the concentration of fibrous aerosols inside buildings covered with asbestos cement plates in four villages. As a result, in the case of long fibers (L > 5 µm), the average concentration was 850 fiber/m^3^, which was three times higher than that of 280 f/m^3^ in the control group. These concentrations can increase the risk of lung cancer in residents of buildings containing asbestos by 10^−6^~10^−5^ and the risk of malignant mesothelioma by 10^−5^~10^−4^ [24].

In our study, we analyzed the data using ELCR, which is the risk of cancer death that exceeds the “natural” background risk resulting from a lifetime exposure to carcinogens [25]. It is a useful means to predict the frequency and severity of carcinogenic effects in exposed populations. EPA classifies ELCR as low risk if it is less than 10^−6^, medium risk if it is 10^−6^ to 10^−5^, and high risk if it is 10^−5^ to 10^−4^ [20]. Two previous Korean studies have measured the risk of carcinogenesis in residents of asbestos-cement slate houses. In the study of Jeong et al. [5], ELCR was found to be at the level of 3.5 × 10^−5^ to 1.5 × 10^−4^, and in the study of Heo et al. [9], the level was 3.21 × 10^−4^ to 2.26 × 10^−5^, and the average level was 1.27 × 10^−4^. The ELCR value of indoor asbestos-cement slate exposure in our study, of 4.81 × 10^−5^, was within the range seen in the two above-mentioned studies.

However, in our study, 89.22% were at moderate risk (1.0 × 10^−5^ ≤ ELCR < 1.0 × 10^−4^), whereas in the study of Jeong et al. [5], 50% of subjects had an ELCR of 1.0 × 10^−4^ or higher, and in the study of Heo et al. [9], 60% of subjects had an ELCR of 1.0 × 10^−4^ or higher. Therefore, there is a possibility that the results of our study may indicate a lower risk estimate.

A possible reason why the risk was lower in our study could be that the asbestos-cement slate roof house residents were generally older, and due to the limitations of the categorical data, the time of the first residence was not accurately determined. Therefore, the TWF may have been underestimated. Further analysis is needed through accurate data collection such as the age of first asbestos-cement slate exposure, asbestos-cement slate-roofed house living period and the length of indoor stay on weekdays and weekends.

In our study, the ELCR of asbestos-cement slate related indoor exposure and occupational dismantling and demolition was found to be medium risk, and the ELCR of residential dismantling and demolition of asbestos-cement slate was less than 10 × 10^−6^, which is considered low risk.

However, in the case of asbestos-related carcinogenicity, there is no threshold, and hence even low risk should not be ignored. When conducting an individual’s risk assessment, it is reasonable to determine the carcinogenic risk based on the exposure period and lifetime exposure taking into account other exposures such as occupational, non-occupational, and environmental exposure as well.

The limitations of this study include difficulty in the comprehensive classification of exposure scenarios due to insufficient literature on the measurement of asbestos-cement slate-related asbestos concentrations. Representativeness was also limited due to the insufficient number of samples included in the pooled analysis for each scenario. Most of the literature analyzed airborne asbestos fiber by Phase-Contrast Microscopy (PCM) rather than Transmission Electron Microscopy (TEM), and the result appears to be non-specific to asbestos. In addition, in the process of converting the geometric mean to the arithmetic mean, outliers occurred that could lead to inaccuracies. Hence, the results excluding outliers are also described. In the risk assessment, it was difficult to accurately evaluate the exposure because the health effect survey data includes only categorical results.

To accurately confirm the exact time and period of residence of the subjects in the asbestos-cement slate roof dense area, we checked the certificate of residence register. However, inaccuracies could creep into ascertaining when asbestos-cement slate roofs were used because this had to rely on the subjects’ memories. It can be inferred by checking the “A register of building” or inquiring about the production schedule of asbestos-cement slate production companies in future studies. In our study, there were no differences in cumulative asbestos exposure levels and ELCR between men and women between indoor exposure and residential asbestos-cement slate roof repair or dismantling. In general, it can be inferred that housewives spend more time indoors and men do more asbestos-cement slate roof repairs, so it may be difficult to understand why there is no difference in asbestos exposure between the sexes. However, this is because when we calculated TWF, the average indoor living time for Korean adults was estimated without discriminating between genders, and since the survey only asked if they had ever repaired their own asbestos-cement slate roof, specific information on the family members who actually spent more time on repairs is not represented. In follow-up studies, it is necessary to scrutinize and re-analyze individual indoor dwelling time and time spent on asbestos-cement slate repair. In addition, there were some cases where we could not find the accurate IUR value so we used an approximate estimate, therefore this could cause a deviation from the actual results.

The asbestos-cement slate roof has been used as a construction material worldwide in the past. Despite the movement to restrict the use of asbestos for decades, the amount of use is still high in developing countries. As the latency period between asbestos exposure and consequent health problems is about 40 years, the burden of asbestos-related diseases has become a worldwide problem nowadays. Therefore, this study suggested representative values of asbestos-cement slate-related asbestos exposure concentrations and ELCR in Korean residences for the first time to confirm the health effects of residential asbestos-cement slate roofs. Through the results of this study, it is possible to predict the frequency or severity of carcinogenic effects of asbestos in “only slate exposed group” people. Furthermore, if there are additional asbestos exposure routes other than residency, it can be helpful to confirm the asbestos exposure intensity by accurately evaluating the lifetime asbestos exposure.

## 5. Conclusions

In our study, the representative values of asbestos exposure concentrations in asbestos-cement slate-roofed houses were calculated, and the carcinogenic risk to residents of such houses was quantitatively evaluated to establish a criterion for an objective and rational evaluation method for asbestos-cement slate-related asbestos exposure intensity. As a result of this study, the representative value of the indoor exposure concentration related to asbestos-cement slate was 0.0032 f/cc on average, and the representative value of the exposure related to asbestos-cement slate dismantling and demolition was 0.0034 f/cc. In addition, the ELCR of asbestos-cement slate-related indoor exposure and occupational dismantling and demolition was found to be of medium risk, and the ELCR of residential dismantling and demolition of asbestos-cement slate was less than 10 × 10^−6^, indicating that the risk was low. However, since there is no threshold for carcinogenicity related to asbestos, this should not be ignored even if the risk appears low, and it would be reasonable to calculate the carcinogenic risk based on total lifetime exposure. More studies on asbestos exposure scenarios and the scope of similar exposure groups through additional data collection and further analysis of risk are needed.

## Figures and Tables

**Table 1:** Characteristics of Korean studies included in this review where the asbestos concentration was presented as f/cc.

Study	Study Year (s)	Number of Samples	GM (±GSD) *	AM (±ASD) **	Min	Max	Analytic Method	Building Construction Year or Type of Exposure
Kim et al. [10]	2008	9	0.0030 (±1.3960)	-	0.0020	0.0060	PCM ***	1960s
		2	0.0010 (±1.0000)	-	0.0010	0.0010	PCM ***	1970s
		8	0.0290 (±7.4040)	-	0.0040	0.2310	PCM ***	1980s
		8	0.0050 (±1.4690)	-	0.0030	0.0100	PCM ***	1990s
Jang et al. [11]	2013	18	0.0001 (±0.04879)	0.0019 (±0.0027)	N.D.	0.0085	PCM ***	<1979
		24	0.00006 (±0.04704)	0.0012 (±0.0015)	N.D.	0.0066		1980–1985
		18	0.0003 (±0.04025)	0.0025 (±0.0026)	N.D.	0.0082		≥1986
Kim et al. [12]	2016	36	0.0024 (Not described)	0.0026 (±0.0011)	0.0008	0.0051	PCM ***	≤1980
		36	0.0027 (Not described)	0.0030 (±0.0013)	0.0012	0.0058		≥1981
Heo et al. [9]	2017	30	0.0027 (±0.0007)	-	0.0012	0.0040	PCM ***	≤1969
		30	0.0022 (±0.0007)	-	0.0082	0.0037		≤1970
		30	0.0024 (±0.0007)	-	0.0008	0.0039		Indoor
		30	0.0025 (±0.0008)	-	0.0012	0.0040		outdoor
Jeong et al. [5]	2013	6	0.0022 (±0.00057)	-	0.0016	0.0031	PCM ***	Indoor
		6	0.0016 (±0.00065)	-	0.0008	0.0027		outdoor
Choi et al. [13]	2001	23	0.047 (±1.658)	-	0.018	0.117	PCM ***	Roof removal
Kim et al. [14]	2009	24	0.002 (±1.956)	-	0.001	0.010	PCM ***	Slate roof
Cho et al. [15]	2013	253	0.002 (±0.004)	-	0.001	1.143	PCM ***	During the process
		316	0.001 (±0.001)	-	0.001	0.009		After the process

* Geometric mean (±Geometric standard deviation); ** Arithmetic mean (±Arithmetic standard deviation); *** Phase-contrast microscopy.

**Table 2:** Asbestos exposure level by each asbestos-cement slate exposure scenario according to the pooled analysis.

Exposure Scenario	Number of Samples	Arithmetic Mean (f/cc)	Arithmetic Standard Deviation	Min	Max
Construction Year					
≤1980	95	0.0025	2.27 × 10^−7^	Not detected	0.0085
>1980	94	0.0207	4.40 × 10^−3^	Not detected	0.0231
(Except outlier)	86	0.0026	1.59 × 10^−6^	Not detected	0.0100
Indoor or Outdoor					
Indoor	10	0.0032	1.73 × 10^−6^	0.0006	0.0067
Outdoor	10	0.0015	2.70 × 10^−8^	0.0002	0.0027
Occupational asbestos-cement slate roof repair or dismantling	616	0.0034	6.62 × 10^−7^	0.0010	1.1430
Residential asbestos-cement slate roof repair or dismantling (estimated)	-	1.83 × 10^−5^	-	-	-

**Table 3:** The characteristics of the asbestos-cement slate-exposed group.

Variable		Number of Subjects	%
No exposure to asbestos other than asbestos-cement slate		575	46.8
Type of asbestos-cement slate exposure	Slate roof only	929	75.65
	Slate roof or slate concentrated area	299	24.35
Exposure period to asbestos-cement slate (years)	≤4	138	11.24
	5~9	148	12.05
	10~19	201	16.37
	≥20	741	60.34
Experience in asbestos-cement slate repair	Absent	866	70.52
	Present	362	29.48
Asbestos-cement slate repair period (years)	None	902	73.45
	≤4	200	16.29
	5~9	55	1.77
	10~19	26	0.84
	≥20	45	1.45
Total		1228	100.00

**Table 4:** Cumulative exposure level to asbestos by each asbestos-cement slate exposure scenario.

Indoor Exposure		Number of Subjects (%)	AM * (f/cc)	ASD **	95% CI ***	
Asbestos cumulative exposure level		575 (100)	0.044	0.014	0.043	0.045
Age	≤29	2 (0.35)	0.017	0.019	−0.152	0.186
	30~39	14 (2.43)	0.024	0.018	0.013	0.034
	40~49	34 (5.91)	0.037	0.016	0.031	0.043
	50~59	103 (17.91)	0.039	0.016	0.036	0.042
	60~69	141 (24.52)	0.045	0.014	0.042	0.047
	≥70	281 (48.87)	0.047	0.011	0.046	0.048
Sex	Male	215 (37.39)	0.044	0.014	0.042	0.046
	Female	360 (62.61)	0.044	0.014	0.042	0.045
**Occupational asbestos-cement slate roof repair or dismantling**		**Number of subjects (%)**	AM * (f/cc)	ASD **	95% CI ***	
Asbestos cumulative exposure level		575 (100)	0.047	0.015	0.045	0.048
Age	≤29	2 (0.35)	0.018	0.020	−0.161	0.198
	30~39	14 (2.43)	0.025	0.019	0.014	0.036
	40~49	34 (5.91)	0.039	0.017	0.033	0.045
	50~59	103 (17.91)	0.042	0.016	0.038	0.045
	60~69	141 (24.52)	0.048	0.015	0.045	0.050
	≥70	281 (48.87)	0.050	0.012	0.049	0.052
Sex	Male	215 (37.39)	0.047	0.015	0.045	0.049
	Female	360 (62.61)	0.047	0.015	0.045	0.048
**Residential asbestos-cement slate roof repair or dismantling**		**Number of subjects (%)**	AM * (f/cc)	ASD **	95% CI ***	
Asbestos cumulative exposure level		575 (100)	0.000251	7.97 × 10^−5^	0.000245	0.000258
Age	≤29	2 (0.35)	9.95 × 10^−5^	0.000108	−0.000870	0.001067
	30~39	14 (2.43)	0.000135	0.000100	7.67 × 10^−5^	0.000193
	40~49	34 (5.91)	0.000212	9.26 × 10^−5^	0.000180	0.000245
	50~59	103 (17.91)	0.000224	8.88 × 10^−5^	0.000206	0.000241
	60~69	141 (24.52)	0.000256	7.85 × 10^−5^	0.000243	0.000269
	≥70	281 (48.87)	0.00027	6.24 × 10^−5^	0.000263	0.000277
Sex	Male	215 (37.39)	0.000251	8.04 × 10^−5^	0.000240	0.000262
	Female	360 (62.61)	0.000251	7.94 × 10^−5^	0.000243	0.000259

* Arithmetic mean; ** Arithmetic standard deviation; *** Confidence interval.

**Table 5:** ELCR by each asbestos-cement slate exposure scenario.

Indoor Exposure		Number of Subjects (%)	AM ** (f/cc)	ASD ***	95% CI ****	
ELCR *		575 (100)	4.81 × 10^−5^	3.53 × 10^−5^	4.52 × 10^−5^	5.10 × 10^−5^
Age	≤29	2 (0.35)	8.70 × 10^−5^	9.84 × 10^−5^	−0.0008	0.000971
	30~39	14 (2.43)	9.51 × 10^−5^	7.84 × 10^−5^	4.98 × 10^−5^	0.00014
	40~49	34 (5.91)	9.68 × 10^−5^	4.86 × 10^−5^	7.99 × 10^−5^	0.000114
	50~59	103 (17.91)	8.20 × 10^−5^	3.85 × 10^−5^	7.45 × 10^−5^	8.95 × 10^−5^
	60~69	141 (24.52)	4.77 × 10^−5^	1.55 × 10^−5^	4.52 × 10^−5^	5.03 × 10^−5^
	≥70	281 (48.87)	2.73 × 10^−5^	5.2 × 10^−5^	2.67 × 10^−5^	2.79 × 10^−5^
Sex	Male	215 (37.39)	5.27 × 10^−5^	4.07 × 10^−5^	4.72 × 10^−5^	5.81 × 10^−5^
	Female	360 (62.61)	4.53 × 10^−5^	3.14 × 10^−5^	4.21 × 10^−5^	4.86 × 10^−5^
**Occupational asbestos-cement slate roof repair or dismantling**		**Number of subjects (%)**	AM ** (f/cc)	ASD ***	95% CI ****	
ELCR		575 (100)	5.11 × 10^−5^	3.75 × 10^−5^	4.80 × 10^−5^	5.41 × 10^−5^
Age	≤29	2 (0.35)	9.24 × 10^−5^	0.0001	−0.0008	0.0010
	30~39	14 (2.43)	0.0001	8.33 × 10^−5^	5.29 × 10^−5^	0.0001
	40~49	34 (5.91)	0.0001	5.17 × 10^−5^	8.48 × 10^−5^	0.0001
	50~59	103 (17.91)	8.71 × 10^−5^	4.09 × 10^−5^	7.91 × 10^−5^	9.51 × 10^−5^
	60~69	141 (24.52)	5.07 × 10^−5^	1.65 × 10^−5^	4.80 × 10^−5^	5.35 × 10^−5^
	≥70	281 (48.87)	2.90 × 10^−5^	5.49 × 10^−5^	2.83 × 10^−5^	2.96 × 10^−5^
Sex	Male	215 (37.39)	5.60 × 10^−5^	4.33 × 10^−5^	5.01 × 10^−5^	6.18 × 10^−5^
	Female	360 (62.61)	4.81 × 10^−5^	3.33 × 10^−5^	4.47 × 10^−5^	5.16 × 10^−5^
**Residential asbestos-cement slate roof repair or dismantling**		**Number of subjects (%)**	AM ** (f/cc)	ASD ***	95% CI ****	
ELCR *		575 (100)	2.75 × 10^−7^	2.02 × 10^−7^	2.58 × 10^−7^	2.92 × 10^−7^
Age	≤29	2 (0.35)	4.98 × 10^−7^	5.63 × 10^−7^	−4.56 × 10^−6^	5.56 × 10^−6^
	30~39	14 (2.43)	5.44 × 10^−7^	4.48 × 10^−7^	2.85 × 10^−7^	8.03 × 10^−7^
	40~49	34 (5.91)	5.54 × 10^−7^	2.78 × 10^−7^	4.57 × 10^−7^	6.51 × 10^−7^
	50~59	103 (17.91)	4.69 × 10^−7^	2.20 × 10^−7^	4.26 × 10^−7^	5.12 × 10^−7^
	60~69	141 (24.52)	2.73 × 10^−7^	8.88 × 10^−8^	2.58 × 10^−7^	2.88 × 10^−7^
	≥70	281 (48.87)	1.56 × 10^−7^	2.96 × 10^−8^	1.52 × 10^−7^	1.59 × 10^−7^
Sex	Male	215 (37.39)	3.01 × 10^−7^	2.33 × 10^−7^	2.70 × 10^−7^	3.33 × 10^−7^
	Female	360 (62.61)	2.59 × 10^−7^	1.79 × 10^−7^	2.41 × 10^−7^	2.78 × 10^−7^

* Excess Lifetime Cancer Risk; ** Arithmetic mean; *** Arithmetic standard deviation; **** Confidence Interval.

**Table 6:** ELCR in indoor slate exposure.

ELCR *	Number of Subjects (%)	Risk Evaluation
<10 × 10^−6^	27 (4.70)	Low
10 × 10^−6^ to 10 × 10^−5^	513 (89.22)	Moderate
10 × 10^−5^ to 10 × 10^−4^	35 (6.09)	High

* Excess Lifetime Cancer Risk.

## Data Availability

Not applicable.

## References

[1] Kang D. (2009). Health effects of environmental asbestos exposure. J. Environ. Health Sci..

[2] Dichicco M.C., De Bonis A., Mongelli G., Rizzo G., Sinisi R. (2017). μ-Raman spectroscopy and X-ray diffraction of asbestos’ minerals for geo-environmental monitoring: The case of the southern Apennines natural sources. Appl. Clay Sci..

[3] Ministry of Environment (MOE) (2009). A Comprehensive Survey for Asbestos Management (Building Asbestos Management Guidelines). http://me.go.kr/home/file/readDownloadFile.do;jsessionid=kd5-JxE6RvBcDmfRsGuUEBHi.mehome1?fileId=6010&fileSeq=1.

[4] Paik D.M., Paik N.W., Choi J.D., Son M.A., Im J.G., Lee W.J., Moon Y.H., Park J.S., Choi B.S. (1995). Prevalence of asbestosis in Korean asbestos industry. Korean J. Occup. Environ. Med..

[5] Jeong J.W., Cho S., Park G.T., Lee S.J. (2013). Health risk assessment and evaluation of asbestos release from asbestos-cement slate roofing buildings in Busan. J. Environ. Sci. Int..

[6] Koo J.W., Kim H.R. (2009). Occupational and environmental asbestos exposure in Korea. J. Korean Med. Assoc..

[7] Ministry of Environment (MOE) (2009). Comprehensive for Asbestos Management. http://me.go.kr/home/file/readDownloadFile.do;jsessionid=Gc5u1mpDGC1SYD4tQD9eLn6E.mehome1?fileId=6253&fileSeq=1.

[8] Busan Metropolitan City Institute of Health & Environment (BIHE) (2013). The 2013 report of total inspection for slate roofing in Busan. Annu. Rep. Busan Metrop. City Inst. Health Environ..

[9] Heo E.H., Jang B.K., Park H.G., Won J.S., Ryu J.W., Jung W.C., Lee J.H., Son B.S. (2017). Concentration of airborne asbestos fiber in indoor and outdoor environment of a slate roofing house, and health risk assessment. J. Odor Indoor Environ..

[10] Kim J.Y. (2008). Exposure Level to Airborne Asbestos Fibers and the Effecting Factors at Building Demolition Sites. Graduate School Thesis.

[11] Jang B.K., Ryu J.Y., Tak H.W., Song S.J., Lee J.H., Lee G.H., Choi J.H. (2013). Asbestos Concentrations in Ambient Air and Drained Rainwater from Slate Roofing by Construction Year and Roof Area. J. Korean Soc. Occup. Environ. Hyg..

[12] Kim Y.J. (2016). Asbestos Concentrations and Size Distribution in Ambient air Around Slate Roofing by Wind Direction. Graduate School Thesis.

[13] Choi C.G., Kim C.N., Lim N.G., Roh Y.M., Roh J.H. (2002). Exposure level of releasing asbestos during building destruction work. J. Korean Soc. Occup. Environ. Hyg..

[14] Kim J.Y., Lee S.K., Lee J.H., Lim M.H., Kang S.W., Phee Y.G. (2009). A study on the factors affecting asbestos exposure level from asbestos abatement in building demolition sites. J. Korean Soc. Occup. Environ. Hyg..

[15] Cho Y.J. (2012). A Study on the Airborne Asbestos Concentration and Health Management Status of the Asbestos Abatement and Removal Working Area. Graduate School Thesis.

[16] Kang D., Kim Y.Y., Shin M.S., Lee M.S., Bae H.J., Kim S.Y., Kim Y.K. (2018). Relationships of lower lung fibrosis, pleural disease, and lung mass with occupational, household, neighborhood, and slate roof-dense area residential asbestos exposure. Int. J. Environ. Res. Public Health.

[17] US Environmental Protection Agency (US EPA) (2008). Framework for Investigating Asbestos-contaminated Superfund Sites (OSWER Directive #9200, 0-68). https://semspub.epa.gov/work/HQ/175329.pdf.

[18] Yoon H.J., Shuai J.P., Kim T.S., Seo J.G., Jung D.Y., Ryu H.S., Yang W.H. (2017). Microenvironmental time-activity patterns of weekday and weekend on Korean adults. J. Odor Indoor Environ..

[19] US Environmental Protection Agency (US PEA), Intergrated Risk Information System (IRIS) (1986). Chemical Assessment Summary (Asbestos; CASRN 1332-21-4). Quantitative Estimate of Carcinogenic Risk from Inhalation Exposure. https://iris.epa.gov/static/pdfs/0371_summary.pdf.

[20] US Environmental Protection Agency (US PEA) (1987). Asbestos Laws and Regulations. Asbestos-Containing Materials in Schools Rule (40 CFR Part 763, Subpart E). https://www.govinfo.gov/content/pkg/CFR-2011-title40-vol31/pdf/CFR-2011-title40-vol31-part763-subpartE.pdf.

[21] US Environmental Protection Agency (US PEA) (2005). Asbestos Exposure and Human Health Risk Assessment, Asbestos Air Sampling, Conducted September 27th through 29th, 2005, Clear Creek Management Area, Califonia; Adult and Child Exposure. https://archive.epa.gov/region9/toxic/web/pdf/ccma_hhra-tech-memo09-05.pdf.

[22] Phanprasit W., Sujirarat D., Chaikittiporn C. (2009). Health risk among asbestos cement sheet manufacturing workers in Thailand. J. Med. Assoc. Thai..

[23] Joob B., Wiwanitki B., Viroj W. (2019). Increased chances of developing cancer due to inhalation of asbestos from roof tile. Egypt J. Chest Dis. Tuberc..

[24] Pastuszka J.S. (2009). Emission of airborne fibers from mechanically impacted asbestos-cement sheets and concentration of fibrous aerosol in the home environment in Upper Silesia, Poland. J. Hazard. Mater..

[25] Freni S.C. (1987). Application of Estimated Excess Lifetime Cancer Risk in Field Situations. Uncertainty in Risk Assessment, Risk Management, and Decision Making.

